# 246. Life-Cycle Emissions Associated with Unnecessary Days of IV Ceftriaxone Therapy

**DOI:** 10.1093/ofid/ofaf695.089

**Published:** 2026-01-11

**Authors:** Anshel Kenkare, Mario Escoriza Gonzalez, Rahee Nerurkar, Kelsie Cowman, Priya Nori

**Affiliations:** Albert Einstein College of Medicine/Montefiore Medical Center - - New York, NY, Long Island City, NY; Albert Einstein College of Medicine/Montefiore Medical Center, Bronx, New York; Albert Einstein College of Medicine/Montefiore Medical Center - - New York, NY, Long Island City, NY; Montefiore Medical Center, Bronx, New York; Montefiore Health System, Bronx, NY

## Abstract

**Background:**

The healthcare sector is responsible for almost 10% of U.S. carbon emissions. Stewardship ensures guideline-concordant prescribing and reduces waste. Guidelines for urinary tract and respiratory tract infections recommend seven or fewer days of antibiotic therapy, but hospitalized patients often receive longer courses. A recent study measured the life-cycle emissions associated with the inpatient administration of intravenous (IV) ceftriaxone, which includes not only the emissions associated with administration and disposal of the IV packaging and tubing, but also the water used to deliver the drug and the waste-associated emissions from gloves and other consumables. This study aimed to quantify IV ceftriaxone courses exceeding seven days, and the emissions associated with the extra days of therapy (DOT), both as an antimicrobial and environmental stewardship target.

**Methods:**

All IV ceftriaxone DOT outside critical care units (including the emergency department and medical-surgical units) at Montefiore Medical Center with a respiratory or urinary tract infection indication were pulled from the electronic record for courses exceeding 7 days. Using established estimates of life-cycle emissions, we calculated the daily environmental impact associated with inpatient administration of IV ceftriaxone. We then multiplied this by the excess days of therapy administered at our institution to quantify total emissions. Emissions were contextualized using the Environmental Protection Agency calculator.

**Results:**

Between 2023-2024, 1089 DOT of ceftriaxone exceeded 7 days for urinary tract or respiratory tract infections. Antibiotic courses that extended beyond 7 days had an average duration of 9.7 days total. These additional days of therapy correspond to 45,787.8 Kg of CO_2_, which is equivalent to charging 3,701,798 smartphones or using 116,601 gallons of gasoline to fuel a car.

**Conclusion:**

Antibiotic courses of 7 days or shorter for respiratory and urinary tract infections are safe and effective. At our medical center, emissions associated with courses of IV ceftriaxone extending beyond 7 days had a significant carbon footprint. Antimicrobial stewardship efforts to reduce unnecessary days of IV antibiotic therapy can therefore have a beneficial environmental impact as well.

**Disclosures:**

All Authors: No reported disclosures

